# Joseph Warren, M.D.

**Published:** 1887-05-28

**Authors:** Oliver Wendell Holmes


### JOSEPH WARREN, M.D.

Trained in the holy art whose lifted shield
Wards off the darts a never-slumbering foe,
By hearth and wayside lurking, waits to throw,
Oppression taught his helpful arm to wield
The slayer's weapon: on the murderous field
The fiery bolt he challenged laid him low,
Seeking its noblest victim. Even so
The charter of a nation must be sealed!
The healer's brow the hero's honour crowned,
From lowliest duty called to loftiest deed.
Living, the oak-leaf wreath his temples bound;
Dying, the conqueror's laurel was his meed,
Last on the broken ramparts' turf to bleed
Where Freedom's victory in defeat was found.

OLIVER WENDELL HOLMES.

